# Case Report: Right atrial mass arising from the Eustachian valve

**DOI:** 10.3389/fcvm.2023.1268918

**Published:** 2023-11-09

**Authors:** Jalal Jolou, Jérôme Martineau, Hajo Müller, Mustafa Cikirikcioglu, Christoph Huber

**Affiliations:** ^1^ Department of Cardiovascular Surgery, Geneva University Hospitals, Geneva University, Geneva, Switzerland; ^2^ Department of Cardiology, Geneva University Hospitals, Geneva University, Geneva, Switzerland

**Keywords:** Eustachian valve, right atrial mass, Chiari network, intra-cardiac mass, thrombi, cardiac MRI

## Abstract

A mass in the right atrium (RA) is an unusual finding that warrants further investigation. We report the case of a 72-year-old male patient who underwent a Bentall operation with a biological composite graft and closure of patent foramen ovale 18 months prior to his presentation with an incidental new RA mass during follow-up echocardiography. Transesophageal echocardiography and thoracic CT angiography confirmed a right atrial mass attached to the Eustachian valve and additionally revealed a non-occlusive pulmonary embolism in the inferior lobar artery of the left lung. Despite 2 months of anticoagulation treatment, the size of the mass did not decrease. Further MRI imaging showed a central mass enhancement which raised concerns about a tumoral lesion. Following a discussion with the local Heart Team, management with surgical treatment was decided. The intraoperative findings revealed a 2.5 cm × 2.1 cm mass arising from the Eustachian valve and a non-diagnosed Chiari network in the RA. Both were resected and sent for a frozen section procedure which excluded a malignancy. The final histopathological analysis described fibrotic tissues compatible with an organized thrombus. The patient was discharged on postoperative day 7 without any complications. Although imaging studies are useful for the initial and differential diagnosis of RA masses, it is not always possible to get the final diagnosis without surgery. In case of a suspicion of a potentially malignant pathology, surgical exploration and resection are necessary.

## Introduction

A mass in the right atrium (RA) is an unusual finding that deserves further investigation. Nowadays, their incidence has increased due to more frequent imaging studies. We report the case of a 72-year-old male patient who had a right atrial mass in a very unusual localization on the Eustachian valve of the RA.

## Case description

**Figure 1:**
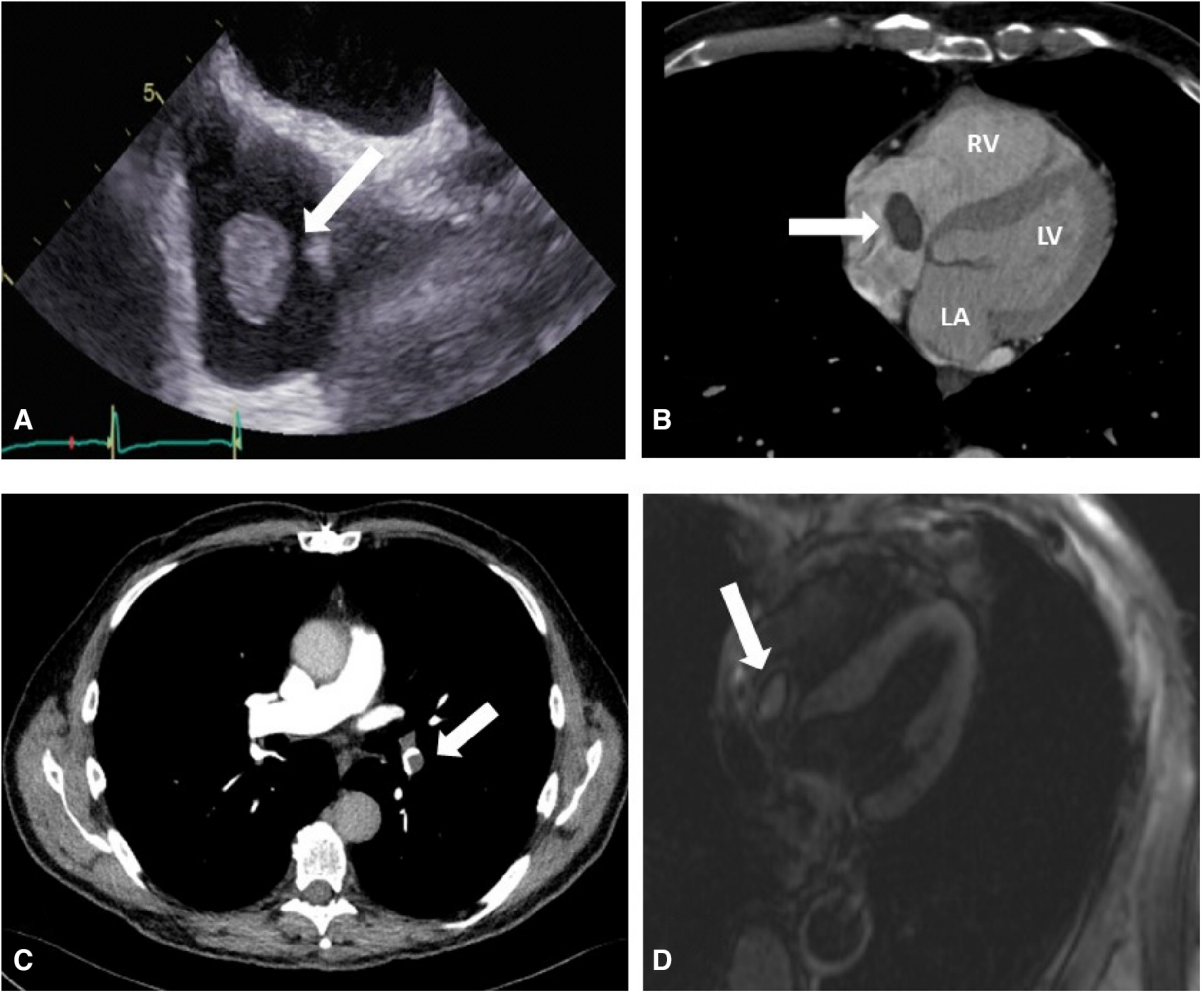
(**A**) Preoperative transesophageal echocardiography shows a mobile right atrial mass (arrow) on the Eustachian valve. (**B**) Preoperative thoracic CT angiography showing the right atrial mass (arrow), right ventricle (RV), left atrium (LA), and left ventricle (LV). (**C**) Preoperative thoracic CT angiography showing pulmonary embolization (PE) on the inferior branch of the left pulmonary artery (arrow). (**D**) MRI imaging that shows a central mass enhancement (arrow) on the Eustachian valve.

A 72-year-old male patient who had undergone a Bentall operation with a biological composite graft and closure of a patent foramen ovale 18 months before, presented with an incidental new RA mass during a follow-up echocardiography. Transesophageal echocardiography and thoracic CT angiography confirmed the presence of the right atrial mass and its location on the Eustachian valve (Figures 1A,B). Moreover, an incidental non-occlusive pulmonary embolism in the left inferior lobe pulmonary artery was diagnosed (Figure 1C). Despite 2 months of oral anticoagulant treatment, the size of the mass did not decrease. An MRI study confirmed the size and location of the mass in the RA and provided further evidence of central enhancement, which raised the suspicion of tumoral lesions (Figure 1D). After consulting with the local Heart Team, the decision to proceed with surgical treatment was made in order to prevent pulmonary embolization and obtain a definite diagnosis of this RA mass.

Under general anesthesia, we proceeded with femoral arterial and venous cannulation, followed by the initiation of cardiopulmonary bypass. We then performed a re-sternotomy and carefully released pericardial adhesions. A second venous cannula was inserted into the superior vena cava. The operation proceeded under normothermic cardiopulmonary bypass, and we operated on the beating heart through a right atriotomy.

**Figure 2:**
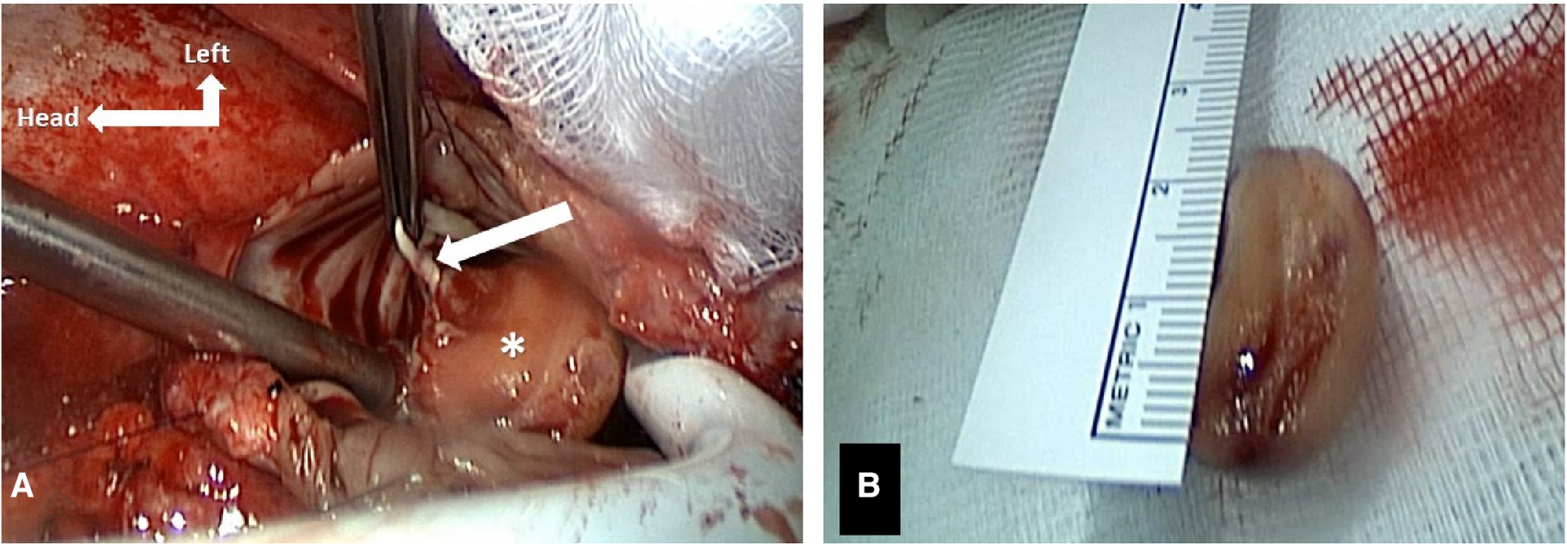
(**A**) Intraoperative findings of the right atrial mass attached to the Eustachian valve (arrow). (**B**) Intraoperative resected mass measuring approximately 2.5 cm × 2.1 cm.

The intraoperative findings revealed a 2.5 cm × 2.1 cm mass (Supplementary Video S1; Figures 2A,B) arising from the Eustachian valve and a preoperatively undiagnosed Chiari network in RA. Both structures were resected and sent for a frozen section procedure, which excluded a malignancy. The final histopathological analysis described fibrotic tissues compatible with an organized thrombus. The patient was discharged on postoperative day 7 without any complications.

## Discussion

The present case describes a right atrial thrombotic mass attached to a very unusual structure, the Eustachian valve, which was discovered during a routine follow-up 18 months after his initial heart surgery which was performed with aorto-bicaval cannulation. During the resection of the RA mass, an extensive and unreported Chiari network was found and removed simultaneously.

The Eustachian valve (EV) is localized around the orifice of the inferior vena cava and right atrial junction. It is a remnant of the intrauterine fetal circulation that helps in directing oxygenated blood from the placenta into the left atrium through the right atrium and patent foramen ovale (1). It usually regresses during childhood. A persistent EV is a frequent finding in patients with a patent foramen ovale (2), as observed in the current reported case.

The Chiari network is occasionally seen in the RA near the opening of the inferior vena cava and the coronary sinus. First described by Hans Chiari in 1897, its prevalence has been variably reported to range between 2% and 13.6% (3–5). It may be associated with thrombi formation (6). It is a fenestrated, net-like structure and akin to the Eustachian valve, represents an embryonic remnant of the sinus venosus valve. The Chiari network is often incidentally diagnosed and may create turbulent flow and cause repetitive pulmonary thromboembolism.

In the presented case, the formation of the thrombus on the EV may be related to the presence of a Chiari network. Another possibility of the formation of the thrombus could be explained by endothelial trauma during the inferior vena cava cannulation in the first operation.

The combination of persistent EV and a Chiari network is a critical combination predisposing to thrombus formation in the RA. Embolization of parts of the RA thrombus is likely to be the source of the diagnosed pulmonary emboli in our patient. Additionally, deep vein thrombosis was ruled out through a dedicated Doppler exam.

Oral anticoagulant treatment is considered the first-line treatment in the presence of intra-cardiac thrombi (7). Because of the size of the RA, the mass did not decrease following 2 months of oral anticoagulant treatment, and suspicion of an intra-cardiac tumor was raised. This suspicion was further supported by central mass contrast uptake observed in the MRI study (Figure 1D). Cardiac MRI can be used to differentiate between intra-cardiac thrombi and tumors (8). Thrombi typically do not show contrast uptake and appear dark on LGE (late gadolinium enhancement), with a surrounding area of high uptake from adjoining blood or myocardium. Conversely, tumors exhibit central contrast uptake and delayed enhancement, as observed in our case, thus prompting surgical treatment. Although cardiac MRI has a high accuracy, in this particular case, it produced a false positive result (9).

## Conclusion

A right atrial thrombotic mass attached to the Eustachian valve discovered during postoperative follow-up is a rare finding and may be misdiagnosed as a potential malignancy. Although imaging studies are useful for the initial and differential diagnosis of RA masses, obtaining a final diagnosis without surgery is not always possible. In case of a suspicion of a malignant process or a possible source of repetitive pulmonary embolism, surgical resection is necessary. Our case highlights the importance of continuous postoperative follow-up including regular imaging exams, even in asymptomatic patients. Swift diagnosis, treatment, and resection may help prevent undesirable outcomes.

## Data Availability

The raw data supporting the conclusions of this article will be made available by the authors, without undue reservation.

## Author’s note

Parts of the content of this manuscript were presented in June 2021 at the Joint Annual Meeting of the Swiss Society of Cardiology (SSC) and the Swiss Society of Cardiac and Thoracic Vascular Surgery (SSCC), and part of its content was included in the Conference Abstract Booklet.

## Supplementary material

Supplementary Video S1 Video of the surgical procedure, showing the right atrial mass attached to the Eustachian valve and its removal.
